# Molecular Phylogeny and Morphology Reveal Four Novel Species of *Corynespora* and *Kirschsteiniothelia* (*Dothideomycetes*, *Ascomycota*) from China: A Checklist for *Corynespora* Reported Worldwide

**DOI:** 10.3390/jof9010107

**Published:** 2023-01-12

**Authors:** Jingwen Liu, Yafen Hu, Xingxing Luo, Rafael F. Castañeda-Ruíz, Jiwen Xia, Zhaohuan Xu, Ruqiang Cui, Xugen Shi, Lianhu Zhang, Jian Ma

**Affiliations:** 1 College of Agronomy, Jiangxi Agricultural University, Nanchang 330045, China; 2 Instituto de Investigaciones de Sanidad Vegetal, Calle 110 No. 514 e/5ta B y 5ta F, Playa, Havana 17200, Cuba; 3 Shandong Provincial Key Laboratory for Biology of Vegetable Diseases and Insect Pests, College of Plant Protection, Shandong Agricultural University, Taian 271018, China

**Keywords:** anamorphic *Ascomycota*, *Kirschsteiniotheliales*, morphology, phylogeny, *Pleosporales*, taxonomy

## Abstract

Plant debris are habitats favoring survival and multiplication of various microbial species. During continuing mycological surveys of saprobic microfungi from plant debris in Yunnan Province, China, several Corynespora-like and Dendryphiopsis-like isolates were collected from dead branches of unidentified perennial dicotyledonous plants. Four barcodes, i.e., ITS, LSU, SSU and *tef1-α*, were amplified and sequenced. Morphological studies and multigene phylogenetic analyses by maximum likelihood and Bayesian inference revealed three new *Corynespora* species (*C*. *mengsongensis* sp. nov., *C*. *nabanheensis* sp. nov. and *C*. *yunnanensis* sp. nov.) and a new *Kirschsteiniothelia* species (*K*. *nabanheensis* sp. nov.) within *Dothideomycetes*, *Ascomycota*. A list of identified and accepted species of *Corynespora* with major morphological features, host information and locality was compiled. This work improves the knowledge of species diversity of *Corynespora* and *Kirschsteiniothelia* in Yunnan Province, China.

## 1. Introduction

Hyphomycetes are highly diverse and distributed in terrestrial and freshwater habitats. More than 1500 Hyphomycetes genera and 30,000 species have been recorded worldwide [1,2]. These fungi show distinct morphological features, which often allow for species identification, as DNA sequences have been hitherto unavailable for most genera and species. Given the large amount of hyphomycetes, it is challenging to classify their taxonomic placement based on morphology alone because some of them may belong to the same species or even to different genera. The introduction of molecular phylogenetic analyses led to a better understanding of the heterogenous genera and species and further clarified their taxonomic status. Investigating fungal diversity is an important task in assembling the fungal tree of life (AFToL) [3], which contributes to the knowledge of biological diversity and the exploration and utilization of fungal resources. 

*Corynespora* was established by Güssow [4] with *C. mazei* as the type species. Wei [5] provided a historical review and considered *C. mazei* a synonym of the previously described *Helminthosporium cassiicola* Berk. & M.A. Curtis and transferred the latter species, resulting in the new combination *Corynespora cassiicola* (Berk. & M.A. Curtis) C.T. Wei. This genus is mainly characterized by distinct, determinate or percurrently extending conidiophores and monotretic, integrated, terminal conidiogenous cells that produce solitary or sometimes catenate, distoseptate conidia [6]. To date, 198 epithets for *Corynespora* have been listed in Species Fungorum [7], but many species associated with leaf spots were defined at least partially on the basis of host identity. Siboe et al. [8] provided a synopsis of basic characteristics of 50 accepted *Corynespora* species, but *C. kenyensis* was not discussed. An additional 93 additional species have since been added to the genus [9,10,11,12,13,14], 87 of which are present in two tables in a format similar to that used by Siboe et al. [8,9,13]. However, *C. alternarioides* [15], *C. camagueyensis* [16], *C. garciniae* [17], *C. inornata* [18], *C. mulanjeensis* [19] and *C. obclavata* [20] were not congeneric with the generic characters in producing euseptate or muriform conidia or synnematous conidiophores with polytretic conidiogenous cells and were excluded from *Corynespora* [21,22,23,24,25]. *Corynespora cespitosa* [26], *C*. *endiandrae* [11], *C*. *leucadendri* [10] and *C*. *olivacea* [27] show the main characters of *Corynespora* but were transferred to *Helminthosporium* by Voglmayr and Jaklitsch [28] based on morphological and phylogenetic analyses. “*Corynespora aeria*” [29], “*C*. *ipomoeae*” [30] (Art. F.5.1: no identifier number cited), “*C*. *masseeanum*” [31] (Art. 41.1: lacking a full and direct basionym reference) and *C. ruelliae* [32] (Art. 40.1: without assigning a type) were not validly published based on the rules of the International Code of Nomenclature for Algae, Fungi, and Plants [33]. Thus, *Corynespora* currently contains 129 valid species. Most *Corynespora* species were introduced primarily based on morphology, and only 10 species with DNA sequences have been used for multigene phylogenetic analyses [12]. 

Sivanesan [34] introduced the family *Corynesporascaceae* Sivan. with *Corynesporasca carotae* Sivan. (= *Corynespora calicioidea* (Berk. & Broome) M.B. Ellis) [2] as the type species, and first connected the teleomorph (*Corynesporasca caryotae*) and anamorph (*Corynespora*) state through cultural studies. Rossman et al. [35] recommended using *Corynespora* over *Corynesporasca*, considering its widespread use, priority and number of species. Subsequently, phylogenetic analyses of five gene regions, i.e., SSU, ITS, LSU, *rpb2* and *tef1-α*, revealed that *Corynespora smithii* forms a separate, distant clade, together with the generic type, *C. cassiicola*, and is treated in the monotypic *Corynesporascaceae* in *Pleosporales* [28].

The genus *Kirschsteiniothelia* was erected by Hawksworth [36] with *K*. *aethiops* as the type species and is mainly characterized by superficial to semi-immersed, subglobose to globose, dark brown to black ascomata; cylindrical clavate, bitunicate, spored asci; and brown to dark brown, ellipsoidal, 1(–2)-septate ascospores with or without a mucilaginous sheath [36,37]. The genus has been linked with two anamorph types, viz., Dendryphiopsis-like and Sporidesmium-like, based on phylogenetic analyses [38]. The Dendryphiopsis-like asexual morph is characteristically macronematous, simple or branched at the apex, forming a stipe and head, brown to dark brown conidiophores with monotretic, integrated, terminal or discrete, determinate or percurrently extending conidiogenous cells that produce solitary, acrogenous, euseptate conidia [38]. The Sporidesmium-like asexual morph has macronematous, unbranched conidiophores with monoblastic, integrated, terminal, determinate or irregular extending conidiogenous cells that produce solitary, acrogenous, euseptate conidia with or without a mucilaginous sheath [38]. Based on morphology and molecular data, previous studies have confirmed that *Dendryphiopsis* is the anamorph of *Kirschsteiniothelia* [37,39], and Wijayawardene et al. [40] further demonstrated that *Dendryphiopsis atra* (generic type) is synonymous with *Kirschsteiniothelia atra* and suggested using *Kirschsteiniothelia* rather than *Dendryphiopsis*, considering the requirement for fewer name changes. Subsequently, seven Sporidesmium-like asexual morphs were reported in *Kirschsteiniothelia* [38,41,42,43,44]. 

The early taxonomic placements of *Kirschsteiniothelia* are uncertain. The genus was originally placed in *Pleosporaceae* by Hawksworth [36] and Barr [45] and subsequently assigned to *Pleomassariaceae* by Barr [46] based on asexual morph connection and morphology. Schoch et al. [47] revealed that *K*. *aethiops* (generic type) does not cluster with *Pleosporaceae* in phylogenetic analyses and suggested that *Kirschsteiniothelia* should be transferred to a new family. Schoch et al. [39] further showed that *K. elaterascus* and *K. maritma* cluster within *Mytilinidion* (*Mytilinidiaceae*) and *Morosphaeria* (*Morosphaeriaceae*), respectively, according to phylogenetic analyses [48], and both species were excluded from *Kirschsteiniothelia* by Boonmee et al. [37]. In addition, Boonmee et al. [37] introduced a new family, *Kirschsteiniotheliaceae*, to accommodate taxa grouping with *K. aethiops* based on morphology and phylogenetic analyses. Hernandez-Restrepo et al. [49] proposed the monotypic order *Kirschsteiniotheliales* for *Kirschsteiniotheliaceae* due to its distant relation to other orders in *Dothideomycetes*.

Yunnan Province is located in southwestern China. It lies at 21°09′–29°15′ N and 97°32′–106°12′ E and includes vast territory with distinct climatic characteristics and abundant natural resources. Its average annual temperatures is 12–22 °C, and the total annual precipitation is approximately 1500 mm. Such favorable conditions support more than 18,000 higher plant species (51.6% of China’s total) in this province, resulting in a very wide range of habitats favoring the growth of various microbial species. However, its mycobiota, especially microfungi, is poorly understood. During our survey of the taxonomy and diversity of saprobic microfungi in Yunnan Province, a Dendryphiopsis-like fungus and three Corynespora-like fungi were collected on dead branches from terrestrial habitats. Based on multilocus phylogenetic analyses and morphological characteristics, we introduced four novel species of *Corynespora* and *Kirschsteiniothelia* in *Dothideomycetes*. This study broadens our understanding of the diversity of *Corynespora* and *Kirschsteiniothelia* taxa.

## 2. Materials and Methods

### 2.1. Sample Collection, Isolation and Morphology

Samples of dead branches were collected randomly from humid environments and river banks, where there is a deep litter layer comprising rotten softwood, dead branches and decayed leaves of various plants in the forest ecosystems of Yunnan Province. Dead branches are a rich habitat for saprobic hyphomycetes. Samples were placed in Ziploc^TM^ bags for transport to the laboratory, where they were processed and examined as described by Ma et al. [50]. Colonies on decaying wood surface were examined and visually observed with a stereomicroscope (Motic SMZ-168, Xiamen, China) from low (0.75 times) to high (5 times) magnification. Fresh colonies were picked with sterile needles at a stereomicroscope magnification of 5 times, placed on a slide with a drop of lactic acid–phenol solution (lactic acid, phenol, glycerin, sterile water; 1:1:2:1, respectively), then placed under an Olympus BX 53 light microscope fitted with an Olympus DP 27 digital camera (Olympus Optical Co., Tokyo, Japan) for microscopic morphological characterization. The tip of a sterile toothpick dipped in sterile water was used to capture the conidia of the target colony directly from the specimen; the conidia were then streaked on the surface of potato dextrose agar (PDA; 20% potato + 2% dextrose + 2% agar, *w/v*) and incubated in an incubator at 25 °C overnight. The single germinated conidia were transferred to fresh PDA plates [51]. Cultures were grown on PDA and incubated in an incubator at 25 °C for 2 weeks; then, morphological characters, including color, shape and size, were recorded. All fungal strains were stored in 10% sterilized glycerin at 4 °C for further studies. The studied specimens and cultures were deposited in the Herbarium of Jiangxi Agricultural University, Plant Pathology, Nanchang, China (HJAUP). The names of the new taxa were registered in Index Fungorum [2].

### 2.2. DNA Extraction, PCR Amplification and Sequencing

Fungal hyphae were scraped from the surface of colonies growing on PDA plates, transferred to 2 mL safe-lock tubes and ground with liquid nitrogen; then, DNA was extracted using a Solarbio fungal genomic DNA extraction kit (Solarbio, Beijing, China) according to the manufacturer’s instructions. DNA amplification was performed by polymerase chain reaction (PCR) using the respective loci (ITS, SSU, LSU and *tef1-α*). The following primer sets were used for these genes: ITS: ITS5/ITS4; SSU: NS1/NS4 [52]; LSU: 28S1-F/28S3-R [53]; and *tef1-α*: EF1-983F/EF1-2218R [54].

The final volume of the PCR reaction was 25 μL, comprising 1 μL of DNA template, 1 μL of each forward and reward primer, 12.5 μL of 2 × Power Taq PCR MasterMix and 9.5 μL of double-distilled water (ddH_2_O). The PCR thermal cycling conditions of ITS, SSU and LSU were initialized at 94 °C for 3 min, followed by 35 cycles of denaturation at 94 °C for 30 s, annealing at 55 °C for 50 s, elongation at 72 °C for 1 min and a final extension at 72 °C for 10 min before being maintained at 4 °C; the *tef1-α* were initialized at 95 °C for 3 min, followed by 35 cycles of denaturation at 95 °C for 30 s, annealing at 60 °C for 30 s, elongation at 72 °C for 1 min and a final extension at 72 °C for 10 min before being maintained at 4 °C. The PCR products were checked by 1% agarose gel electrophoresis staining with ethidium bromide. Purification and DNA sequencing were carried out at Beijing Tsingke Biotechnology Co., Ltd. China. New sequences generated in this study were deposited in the NCBI GenBank (www.ncbi.nlm.nih.gov, accessed on 10 December 2022; Table 1 and Table 2).

### 2.3. Phylogenetic Analyses

The newly generated sequences, together with other sequences obtained from GenBank (four loci: ITS, LSU, SSU and *tef1-α* (Table 1); three loci: ITS, LSU and SSU (Table 2)), were separately aligned using the MAFFTv.7 [55] online server (http://maffTh.cbrc.jp/alignment/server/, accessed on 23 December 2022) and manually optimized when needed. Phylogenetic analyses were first conducted individually for each locus, then for a combined dataset of these loci. The four ITS, LSU, SSU and *tef1-α* alignment datasets and the three ITS, LSU and SSU alignment datasets were concatenated with Phylosuite software v1.2.2 [56], and absent sequence data in the alignments were treated with a question mark as missing data. Phylosuite software v1.2.2 [56] was used to construct separate phylogenetic trees based on ITS, LSU, SSU and *tef1-α* sequence data, as well as ITS, LSU and SSU sequence data. The concatenated and aligned datasets were analyzed separately using maximum likelihood (ML) and Bayesian inference (BI). The maximum-likelihood phylogenies were inferred using IQ-TREE [57] under an edge-linked partition model for 10000 ultrafast bootstraps [58]. For *Corynespora*, the final tree was selected among suboptimal trees from each run by comparing the likelihood scores using SYM+G4 for ITS, TNe+G4 for LSU+*tef1-α* and K2P+I for the SSU substitution model. Bayesian inference phylogenies were inferred using MrBayes 3.2.6 [59] under a partition model (2 parallel runs, 2000000 generations), in which the initial 25% of sampled data were discarded as burn-in. The best-fit model was SYM+G4 for ITS, GTR+F+G4 for LSU+*tef1-α* and K2P+I for SSU. For *Kirschsteiniothelia*, the final tree was selected among suboptimal trees from each run by comparing the likelihood scores using TIM2e+R3 for ITS+SSU and TN+F+G4 for the LSU substitution model. Bayesian inference phylogenies were inferred using MrBayes 3.2.6 [59] under a partition model (2 parallel runs, 2000000 generations), in which the initial 25% of sampled data were discarded as burn-in. The best-fit model was SYM+G4 for ITS+LSU+SSU. ModelFinder [60] was used to select the best-fit partition model (edge-linked) using the BIC criterion. The trees were viewed in FigTree v. 1.4.4 (http://tree.bio.ed.ac.uk/software/figtree, accessed on 10 December 2022) and further edited in Adobe Illustrator 2021.

## 3. Results

### 3.1. Molecular Phylogeny

**Figure 1:**
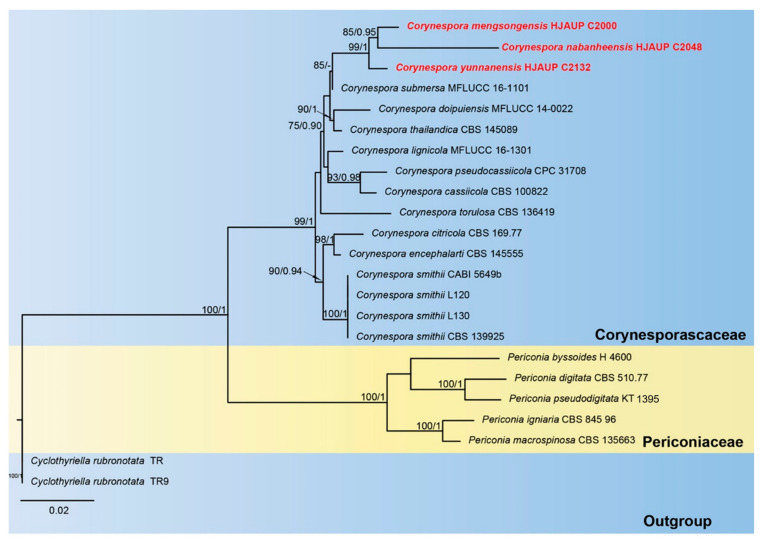
Phylogram of *Pleosporales* based on combined ITS, SSU, LSU and *tef1-α* sequences. ML and BI bootstrap support values above 75% and 0.90 are shown at the first and second position, respectively. The tree is rooted to *Cyclothyriella rubronotata* (TR) and *C. rubronotata* (TR9). Strains from the current study are indicated in red.

The phylogenetic tree (Figure 1) inferred from maximum-likelihood and Bayesian inference analyses based on combined ITS, LSU, SSU and *tef1-α* sequence data consisted of three families (*Corynesporascaceae*, *Periconiaceae* and *Cyclothyriellaceae*). The concatenated sequence matrix comprised 23 sequences with 3147 total characters (the combined dataset, ITS: 1–498, LSU: 499–1348, SSU: 1349–2374, *tef1-α*: 2375–3147), 537 distinct patterns, 375 parsimony-informative sites, 147 singleton sites and 2625 constant sites; *Cyclothyriella rubronotata* (TR) and *C*. *rubronotata* (TR9) were regarded as an outgroup. Maximum-likelihood and Bayesian inference analyses of the combined dataset resulted in phylogenetic reconstructions with largely similar topologies; the best-scoring ML tree is shown in Figure 1. Bootstrap support values for maximum likelihood higher than 75% and Bayesian posterior probabilities greater than 0.90 are shown above the nodes. The best-scoring ML consensus tree (lnL = –8859.832) with ultrafast bootstrap values from ML analyses and posterior probabilities from MrBayes analysis at the nodes is shown in Figure 1. The strains of *Corynespora mengsongensis* form a distinct clade sister to *C*. *nabanheensis* with good statistical support (ML/BI = 85/0.95); *C. yunnanensis* forms a high-support clade (ML/BI = 99/1.00) with the lineage consisting of *C. mengsongensis* and *C*. *nabanheensis*, and they form a sister clade to *C*. *submersa* (ML/BI =85/0.75).

**Figure 2:**
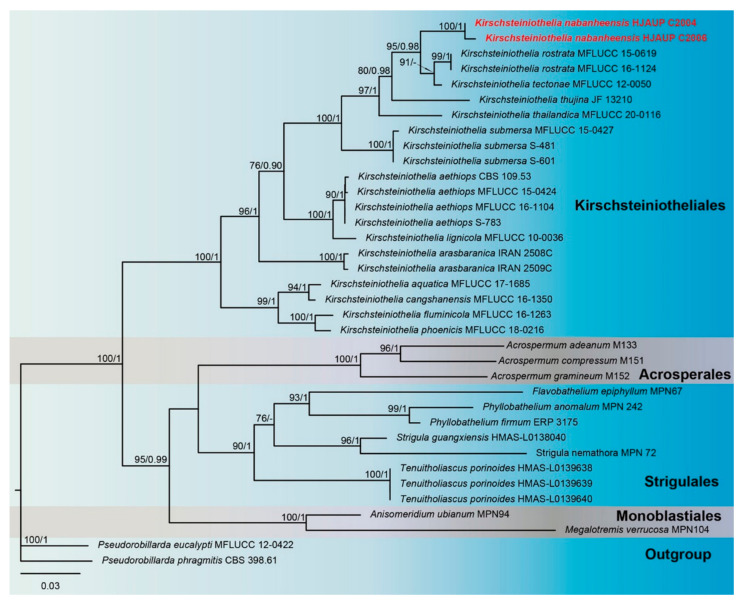
Phylogram of *Kirschsteiniotheliales*, *Acrosperales*, *Strigulales* and *Monoblastiales* based on combined ITS, SSU and LSU sequences. The ML and BI bootstrap support values above 75% and 0.90 are shown at the first and second position, respectively. The tree is rooted to *Pseudorobillarda eucalypti* (MFLUCC 12–0422) and *P. phragmitis* (CBS 398.61). Strains from the current study are indicated in red.

The phylogenetic tree (Figure 2) inferred from maximum-likelihood and Bayesian inference analyses based on combined ITS, LSU and SSU sequence data consisted of four orders (*Acrospermales*, *Kirschsteiniotheliales*, *Monoblastiales* and *Strigulales*). The concatenated sequence matrix comprised 36 sequences with 1260 total characters (combined dataset, ITS: 1–162, LSU: 163–471, SSU: 472–1260), 413 distinct patterns, 253 parsimony-informative sites, 183 singleton sites and 824 constant sites; *Pseudorobillarda eucalypti* (MFLUCC 12–0422) and *P*. *phragmitis* (CBS 398.61) were regarded as an outgroup. Maximum-likelihood and Bayesian inference analyses of the combined dataset resulted in phylogenetic reconstructions with largely similar topologies; the best-scoring ML tree is shown in Figure 2. Bootstrap support values for maximum likelihood higher than 75% and Bayesian posterior probabilities greater than 0.90 are shown above the nodes. The best-scoring ML consensus tree (lnL = –6307.741) with ultrafast bootstrap values from ML analyses and posterior probabilities from MrBayes analysis at the nodes is shown in Figure 2. The strains of *K*. *nabanheensis* form a separate clade closely related to *K*. *thailandica*, *K. thujina*, *K. tectonae* and *K. rostrata*, with strong statistical support (ML/BI = 95/0.98).

### 3.2. Taxonomy

**Figure 3:**
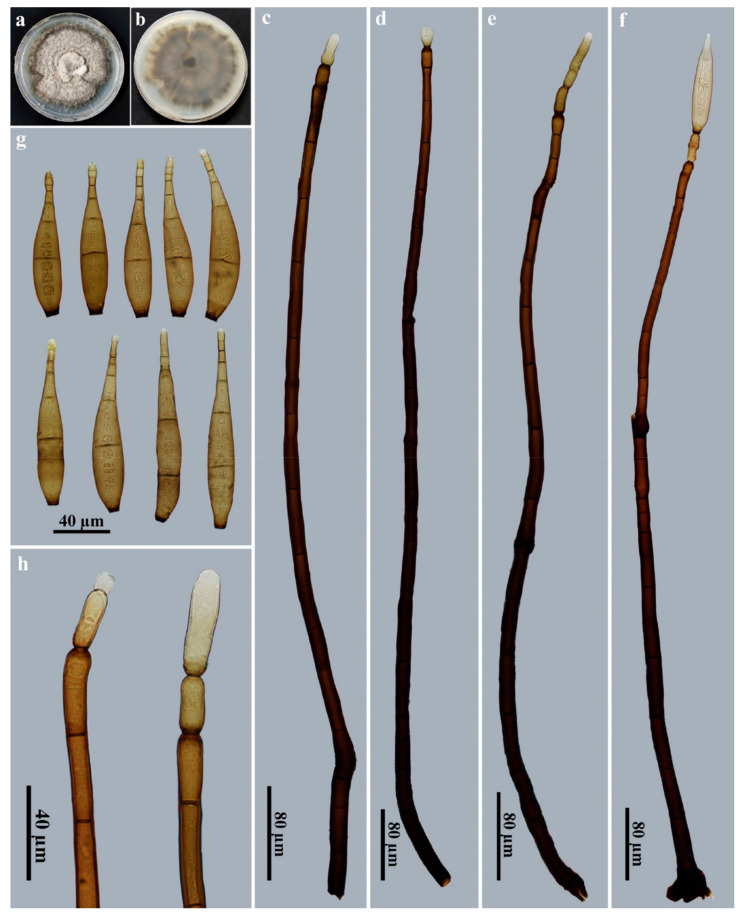
*Corynespora mengsongensis* (HJAUP M2000, holotype). (**a**) Surface of colony after 2 weeks on PDA; (**b**) reverse of colony after 2 weeks on PDA; (**c**–**f**) conidiophores, conidiogenous cells and conidia; (**g**) conidia; (**h**) conidiogenous cells with developing conidia.

*Corynespora mengsongensis* Jing W. Liu & Jian Ma, sp. nov., Figure 3.

Indexfungorum number: IF900076

Etymology: The name refers to Mengsong, the township where the fungus was collected.

Holotype: HJAUP M2000.

Description: Saprobic on decaying wood in terrestrial habitats. **Teleomorph:** undetermined. **Anamorph** (Figure 3): Hyphomycetes. *Colonies* on natural substratum are effuse, brown to dark brown and hairy. *Mycelium* is superficial and immersed, composed of branched, septate, pale brown to brown, smooth-walled hyphae. *Conidiophores* are macronematous, mononematous, unbranched, erect, straight or flexuous, cylindrical, smooth, brown to dark brown and septate, with up to 2 successive cylindrical extensions (267–)746–938 × 12.5–17 μm ($\bar{X}$ = 829 × 14.5 μm, n = 15). *Conidiogenous cells* are integrated, terminal, monotretic, cylindrical, pale brown to brown and smooth, with dimensions of 25–29 × 8.5–10.5 µm ($\bar{X}$ = 27 × 9.4 μm, n = 15). *Conidia* are acrogenous, solitary, obclavate, rostrate, rounded at the apex, straight or slightly curved, 13–18-distoseptate, brown to golden brown and smooth, with dimensions of 96–146 × 16.5–20.5 μm ($\bar{X}$ = 118.5 × 18.5 μm, n = 20), tapering to 3–4 μm near the apex and truncate at the base, with a protuberant dark-brown hilum that is 6–8 μm wide at the base.

Culture characteristics: Colony on PDA reaching 80–88 mm diam. after 2 weeks in an incubator under dark conditions at 25 °C; irregular circular, velvety surface with dense, gray–white mycelia along the entire margin; reverse brown to dark brown.

Material examined: China, Yunnan Province, Xishuangbanna Dai Autonomous Prefecture, Menghai County, Mengsong Township, on dead branches of an unidentified broadleaf tree, 12 July 2021, J.W. Liu (HJAUP M2000, ***holotype***; ex-type culture permanently preserved in a metabolically inactive state, HJAUP C2000).

Notes: Phylogenetic analyses showed that *C*. *mengsongensis* cluster with *C*. *nabanheensis* (Figure 1). BLASTn analysis of *C*. *mengsongensis* (HJAUP C2000^T^) and *C*. *nabanheensis* (HJAUP C2048^T^) shows 90% identity (540/598, 22 gaps) using ITS, 97% identity (559/578, 3 gaps) using LSU and 99% identity (1021/1026, no gaps) using SSU. *Corynespora mengsongensis* are morphologically similar to *C*. *merrilliopanacis* [61], but the latter differ in terms of their longer conidiophores (260–1200 μm), with up to 5 successive cylindrical extensions and longer conidia (130–260 μm) with 12–25 distosepta. Furthermore, *C. mengsongensis* differ from *C*. *nabanheensis*, which have smaller conidiophores (282–528 × 6–8 μm) with 3–4 successive cylindrical extensions and smaller conidia (56–84 × 12–14 μm) with 9–13 distosepta.

**Figure 4:**
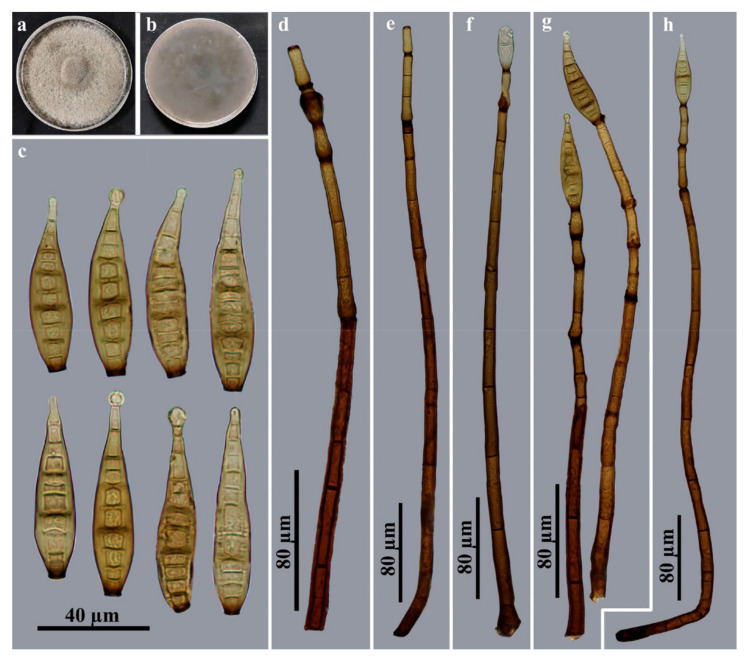
*Corynespora nabanheensis* (HJAUP M2048, holotype). (**a**) Surface of colony after 2 weeks on PDA; (**b**) reverse of colony after 2 weeks on PDA; (**c**) conidia; (**d**,**e**) conidiophores and conidiogenous cells; (**f**–**h**) conidiophores, conidiogenous cells and conidia.

*Corynespora nabanheensis* Jing W. Liu & Jian Ma, sp. nov., Figure 4.

Index Fungorum number: IF900077

Etymology: The name refers to Nabanhe Nature Reserve, the locality where the fungus was collected.

Holotype: HJAUP M2048.

Description: Saprobic on decaying wood in terrestrial habitats. **Teleomorph:** undetermined. **Anamorph** (Figure 4): hHyphomycetes. ***Colonies*** on natural substratum are effuse, brown to dark brown and hairy. Mycelia are superficial and immersed, composed of branched, septate, pale brown to brown, smooth-walled hyphae. *Conidiophores* are macronematous, mononematous, unbranched, erect, straight or flexuous, cylindrical, smooth and brown to dark brown, with 10–17-septate with 3–4 successive cylindrical extensions and dimensions of 282–528 × 6–8 μm ($\bar{X}$ = 388 × 7 μm, n = 15). *Conidiogenous cells* are integrated, terminal, monotretic, cylindrical, pale brown to brown and smooth, with dimensions of 20–32 × 5–8 µm ($\bar{X}$= 25.5 × 6.5 μm, n = 15). *Conidia* are acrogenous, solitary, obclavate, rostrate and straight or slightly curved with 9–13-distoseptate, brown to golden brown, smooth and usually expanded to a rounded shape at the apex, with dimensions of 56–84 × 12–14 μm ($\bar{X}$ = 66.5 × 13 μm, n = 20) and 4–6 μm near the apex and truncated at the base, with a protuberant dark brown hilum that is 4–5 μm wide at the base.

Culture characteristics: Colonies on PDA reach 85–90 mm diam. after 2 weeks in an incubator under dark conditions at 25 °C, with an irregular, circular, velvety surface and dense, gray mycelia along the entire margin; reverse brown to black.

Material examined: China, Yunnan Province, Xishuangbanna Dai Autonomous Prefecture, the Nabanhe National Nature Reserve, on dead branches of an unidentified broadleaf tree, 12 July 2021, J.W. Liu (HJAUP M2048, ***holotype***; ex-type culture permanently preserved in a metabolically inactive state HJAUP C2048).

Notes: Phylogenetic analyses showed that *C*. *nabanheensis* cluster with *C*. *mengsongensis* (Figure 1). BLASTn analysis of *C*. *nabanheensis* (HJAUP C2048^T^) and *C*. *mengsongensis* (HJAUP C2000^T^) shows 90% identity (540/598, 22 gaps) using ITS, 97% identity (559/578, 3 gaps) using LSU and 99% identity (1021/1026, no gaps) using SSU. *Corynespora nabanheensis* are morphologically similar to *C*. *doipuiensis* [12], but the latter differ in terms of their shorter and wider conidiophores (212–426 × 10–15 μm), with fewer successive cylindrical extensions and larger, obconical, guttulate, subhyaline to moderately brown conidia (136–165 × 5–25.5 μm). Furthermore, *C*. *nabanheensis* differ from *C*. *mengsongensis*, which have larger conidiophores (746–938 × 12.5–17 μm), with up to 2 successive cylindrical extensions and larger conidia (96–146 × 16.5–20.5 μm) with 13–18 distosepta.

**Figure 5:**
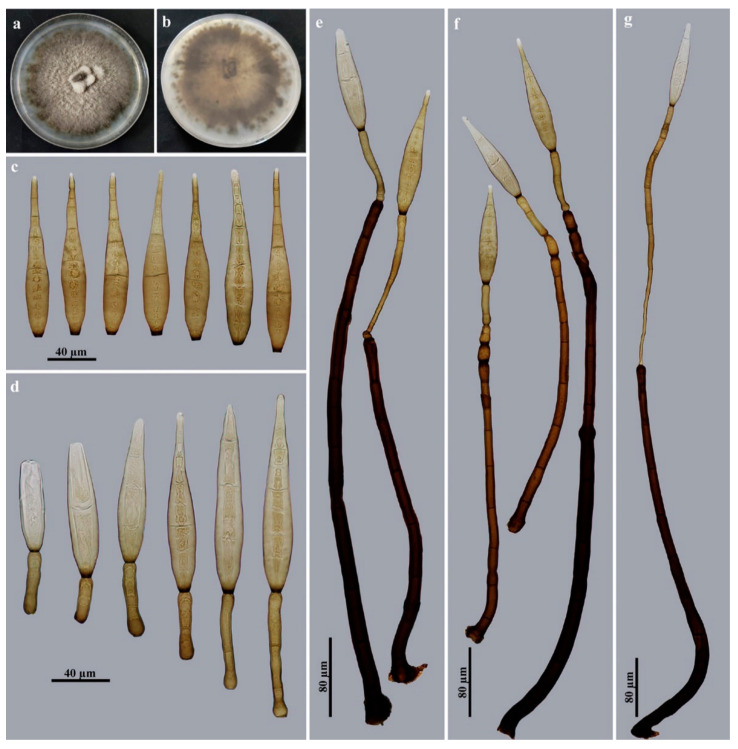
*Corynespora yunnanensis* (HJAUP M2132, holotype). (**a**) Surface of colony after 2 weeks on PDA; (**b**) reverse of colony after 2 weeks on PDA; (**c**) conidia; (**d**) conidiogenous cells and conidia; (**e**–**g**) conidiophores, conidiogenous cells and conidia.

*Corynespora yunnanensis* Jing W. Liu & Jian Ma, sp. nov., Figure 5.

Index Fungorum number: IF900078.

Etymology: The name refers to Yunnan, the province where the fungus was collected.

Holotype: HJAUP M2132.

Description: Saprobic on decaying wood in terrestrial habitats. **Teleomorph:** undetermined. **Anamorph** (Figure 5): Hyphomycete. *Colonies* on natural substratum are effuse, brown to dark brown and hairy. *Mycelia* are superficial and immersed, composed of branched, septate, pale brown to brown, smooth-walled hyphae. *Conidiophores* are macronematous, mononematous, unbranched, erect, straight or flexuous, cylindrical, smooth, septate and brown to dark brown, with 1–4 successive cylindrical extensions and dimensions of 380–844 × 8–16 μm ($\bar{X}$ = 547 × 12.5 μm, n = 15). *Conidiogenous cells* are integrated, terminal, monotretic, cylindrical, pale brown to brown and smooth, with dimensions of 44–120(–332) × 6–8 µm ($\bar{X}$ = 55.5 × 6.5 μm, n = 15). *Conidia* are acrogenous, solitary, obclavate, rostrate, rounded at the apex, straight or slightly curved, 3–16-distoseptate, brown to golden brown and smooth, with dimensions of 80–128 × 16–19 μm ($\bar{X}$ = 117 × 18 μm, n = 25), tapering to 4–8 μm near the apex, and truncated at the base with a protuberant dark brown hilum that is 6–8 μm wide at the base.

Culture characteristics: Colonies on PDA reach 78–85 mm diam. after 2 weeks in an incubator under dark conditions at 25 °C, with an irregular, circular, velvety surface with dense, gray mycelia along the entire margin; reverse brown to black.

Material examined: China, Yunnan Province, Xishuangbanna Dai Autonomous Prefecture, Jinghong City, Gasa Township, on dead branches of an unidentified broadleaf tree, 12 July 2021, J.W. Liu (HJAUP M2132, ***holotype***; ex-type culture permanently preserved in a metabolically inactive state HJAUP C2132).

Notes: Phylogenetic analyses showed that *C*. *yunnanensis* cluster with *C*. *mengsongensis* and *C*. *nabanheensis*, and they form a sister clade to *C*. *submersa* (Figure 1). BLASTn analysis of *C*. *yunnanensis* (HJAUP C2132^T^) and *C*. *mengsongensis* (HJAUP C2000^T^) shows 99% identity (567/569, 2 gaps) using ITS, 98% identity (577/587, 7 gaps) using LSU and 99% identity (1028/1029, no gaps) using SSU. BLASTn analysis of *C*. *yunnanensis* (HJAUP C2132^T^) and *C*. *nabanheensis* (HJAUP C2048^T^) shows 91% identity (518/569, 20 gaps) using ITS, 97% identity (543/558, 1 gap) using LSU and 99% identity (1024/1028, no gaps) using SSU. BLASTn analysis of *C*. *yunnanensis* (HJAUP C2132^T^) and *C*. *submersa* (MFLUCC 16-1101) shows 100% identity (487/487, no gaps) using ITS and 99% identity (544/547, 1 gap) using LSU. *Corynespora yunnanensis* are morphologically similar to *C*. *submersa* [12], but the latter differ by in terms of their shorter and narrower conidiophores (150–370 × 10–12 μm) and larger, catenate conidia (100–150 × 16–24 μm), with 9–13 distosepta. Furthermore, *C*. *yunnanensis* differ from *C*. *mengsongensis*, which have larger conidiophores (746–938 × 12.5–17 μm), with up to 2 successive cylindrical extensions, and larger conidia (96–146 × 16.5–20.5 μm) with 13–18 distosepta, as well as from *C*. *nabanheensis*, which have smaller conidiophores (282–528 × 6–8 μm) and smaller conidia (56–84 × 12–14 μm) with 9–13 distosepta.

**Figure 6:**
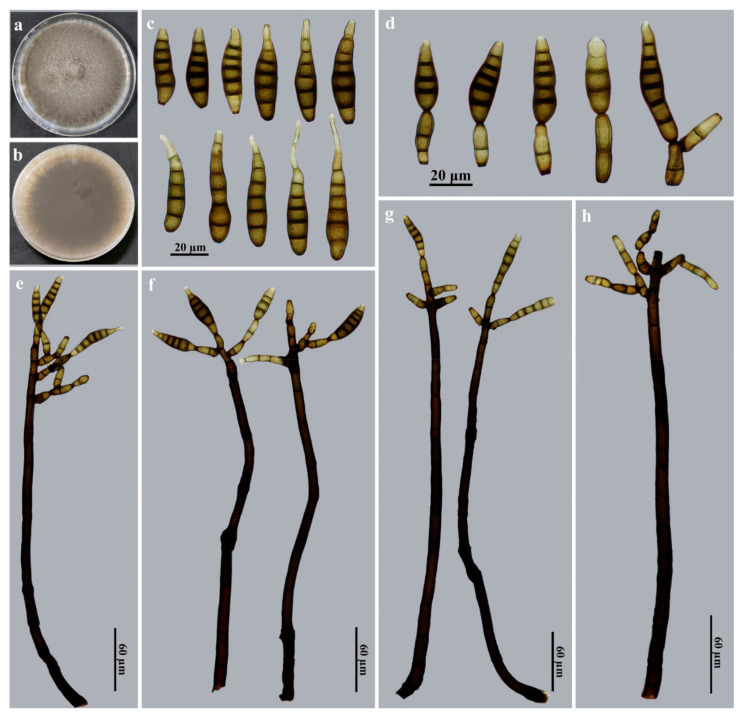
*Kirschsteiniothelia nabanheensis* (HJAUP M2004, holotype). (**a**) Surface of colony after 2 weeks on PDA; (**b**) reverse of colony after 2 weeks on PDA; (**c**) conidia; (**d**) conidiogenous cells and conidia; (**e**–**g**) conidiophores, conidiogenous cells and conidia; (**h**) conidiophores and conidiogenous cells.

*Kirschsteiniothelia nabanheensis* Jing W. Liu & Jian Ma, sp. nov., Figure 6.

Index Fungorum number: IF900082.

Etymology: The name refers to Nabanhe Nature Reserve, the locality where the fungus was collected.

Holotype: HJAUP M2004.

Description: Saprobic on decaying wood in terrestrial habitats. **Teleomorph:** undetermined. **Anamorph** (Figure 6): Hyphomycetes. *Colonies* on natural substratum are effuse, brown to black and hairy. *Mycelia* are superficial and immersed, composed of branched, septate, pale brown to brown, smooth-walled hyphae. *Conidiophores* are macronematous, mononematous, erect, irregular or subscorpioid branched near the apex, solitary, smooth, cylindrical, straight to flexuous, septate and black–brown to brown, with dimensions of (200–)320–588 × 8–12 μm ($\bar{X}$ = 405 × 9.5 μm, n = 15). *Conidiogenous cells* are monotretic, integrated, terminal or intercalary, cylindrical or doliiform, determinate, smooth and brown to dark brown, with dimensions of 20–24 × 4–6 µm ($\bar{X}$ = 22 × 5 μm, n = 15). *Conidia* are acrogenous, solitary, obclavate or fusiform, sometimes rostrate, straight or slightly curved, 3–7-euseptate, dark brown to brown and smooth, with dimensions of 32–112 × 8–12 μm ($\bar{X}$ = 55.5 × 10 μm, n = 25), tapering to 3–4 μm near the apex, with a width of 4–5 μm at the base.

Culture characteristics: Colonies on PDA reach 85–90 mm diam. after 2 weeks in an incubator under dark conditions at 25 °C, with an irregular, circular, velvety surface with dense, gray–brown mycelia along the entire margin; reverse brown to dark brown.

Material examined: China, Yunnan Province, Xishuangbanna Dai Autonomous Prefecture, the Nabanhe National Nature Reserve, on dead branches of an unidentified broadleaf tree, 12 July 2021, J.W. Liu (HJAUP M2004, ***holotype***; ex-type culture permanently preserved in a metabolically inactive state HJAUP C2004 = HJAUP C2006).

Notes: Phylogenetic analyses showed that *K*. *nabanheensis* cluster with *K*. *thailandica*, *K. thujina*, *K. tectonae* and *K. rostrata*, and they form a sister clade to *K*. *submersa* (Figure 2). BLASTn analysis of *K*. *nabanheensis* (HJAUP C2004^T^) and *K*. *rostrata* (MFLUCC 15–0619^T^) shows 85% identity (473/558, 27 gaps) using ITS, 95% identity (519/548, 4 gaps) using LSU and 99% identity (875/886, no gaps) using SSU. BLASTn analysis of *K*. *nabanheensis* (HJAUP C2004^T^) and *K*. *tectonae* (MFLUCC 12–0050^T^) shows 84% identity (458/548, 35 gaps) using ITS and 95% identity (523/548, 4 gaps) using LSU. *Kirschsteiniothelia nabanheensis* are morphologically similar to *K*. *shimlaensis*, but the latter differ in terms of their shorter and wider conidiophores (110–268 × 12–19 μm), and shorter and wider, obovoid, oblong, clavate or cylindrical conidia (41–81 × 13–17.5 μm) with 2–5(–6) eusepta [62]. Furthermore, *K. nabanheensis* differ from *K*. *submersa* which have smaller conidiophores (220–280 × 6–7 μm) and holoblastic conidiogenous cells producing smaller conidia (37.5–51.5 × 8.5–9.5 μm) with 4–6 eusepta [42].

## 4. Discussion

In this study, we collected saprophytic hyphomycetes on dead branches from terrestrial habitats in Yunnan Province, China. Based on the morphomolecular approach, four novel taxa are introduced: *Corynespora mengsongensis* sp. nov., *C*. *nabanheensis* sp. nov., *C*. *yunnanensis* sp. nov. and *Kirschsteiniothelia nabanheensis* sp. nov.

*Corynespora* show high morphological similarity to *Corynesporina*, *Corynesporopsis*, *Hemicorynespora* and *Solicorynespora* in terms of their terminal, monotretic, conidiogenous cells and differ only on the basis of single conidial characteristics (e.g., single or catenate, euseptate or distoseptate, basipetal chain or acropetal chain) [63]. The weak differentiation of these similar genera should only be maintained until sufficient molecular analysis allows for a more phylogenetic classification of genera. In addition, it is challenging to separate *Corynespora* from *Helminthosporium* based on morphology alone, as four *Corynespora* species, *C. caespitosa*, *C. endiandrae*, *C. leucadendri* and *C. olivacea*, were transferred to *Helminthosporium* based on molecular phylogenetic analyses, which led to the genus *Helminthosporium* also meeting the criteria of *Corynespora* [28].

The genus *Corynespora* produces conspicuous morphological features, and its generic type, *C. cassiicola*, is a ubiquitous species, mainly in tropical and subtropical areas, and has been recorded from a wide range of plants [64]. Most *Corynespora* species are known as saprobes and plant pathogens from woody and herbaceous hosts [8,9,13], but occasionally, *C. cassiicola* is also found in nematodes, sponges and human skin [64]. To date, 132 species of *Corynespora* (Table 3) have been be accepted worldwide, whereas four invalid names enclosed in quotation marks are also listed in Table 3. Many species have been identified only based on morphological studies, and only 13 species, including our three new species, have been subjected to molecular phylogenetic analyses. Morphological comparison is important for species identification, but the lack of a large amount of molecular data made it difficult to evaluate previously described *Corynespora* species by molecular methods. Thus, we recommend supplementary sequence data for previously described *Corynespora* species by re-examining their type materials or collecting fresh new specimens and using molecular phylogenetic analyses to evaluate their taxonomic placement as necessary.

Hawksworth [36] established the genus *Kirschsteiniothelia* and regarded *K*. *aethiops* as the type species. Boonmee et al. [37] treated the genus in a new family, *Kirschsteiniotheliaceae*, based on evidence from morphological and phylogenetic analyses. Hernandez-Restrepo et al. [49] raised *Kirschsteiniotheliaceae* to the new order *Kirschsteiniotheliales* in *Dothideomycetes*, although this order does not form a well-supported clade within *Dothideomycetidae* as a sister clade to *Asterinales*; the two orders diverged approximately 221 MYA according to divergence time estimates [65].

Sun et al. [38] accepted five former *Dendryphiopsis* species, *D. arbuscula*, *D. binsarensis*, *D. biseptata*, *D. fascicularis* and *D. goaensis*, in *Kirschsteiniothelia* following the latest treatment of *Dendryphiopsis* by Wijayawardene et al. [40]. However, these five species were invalidly introduced as new combinations in *Kirschsteiniothelia* on the basis of Art. F.5.1 (no identifier number cited) and Art. 41.1 (lacking a full and direct basionym reference) of the International Code of Nomenclature for Algae, Fungi, and Plants [33]. In addition, Sun et al. [38] provided a checklist for 35 *Kirschsteiniothelia* species including the distribution, habitat, host and morphology type of each species, but *K. ebriosa* [66] and *K. vinigena* [66] are not included. Subsequently, Verma et al. [62] described a new species, *K. shimlaensis*, from decaying stump in India.

**Table 3:** Synopsis of conidial characteristics, host information and locality compared across *Corynespora* species.

Species	Conida					Host/Locality	References
	Production	Morphology	Color	Size (µm)	Septation		
*Corynespora acaciae*	Solitary	Obclavate	Dark brown	16–30 × 6–8	1–5	On phyllodes of *Acacia pycnantha*, Australia	[67]
*C. acalyphae*	Solitary	Obclavate, rostrate	Pale brown to brown	85–120 × 9–11	8–16	On dead branches of *Acalypha hamiltoniana*, Indonesia	[68]
*C. achradis*	Solitary or catenate	Obclavate, rostrate	Pale olivaceous brown	60–95 × 6–7	5–10	On leaves of *Achras sapota*, Brunei	[69]
“*C. aeria*”	Solitary	Obclavate	Subhyaline to olivaceous	Up to 350 × 2–5	1–5	Isolated from air, India	[29]
*C. albiziicola*	Solitary	Obclavate, ellipsoid or clavate	Pale olivaceous yellow	20–70.1 × 10–18.5	1–6	On leaves of *Albizia lebbek*, India	[70]
*C. alstoniae*	Solitary or catenate	Cylindrical to obclavate	Subhyaline to light olivaceous	48–154 × 8–21.5	2–15	On leaves of *Alstonia scholaris*, Nepal	[71]
*C. annonacea*	Solitary or catenate	Obclavate to obclavate–cylindrical	Subhyaline to olivaceous brown	25–135 × 10–18	1–10	On living leaves of *Annona squamosa*, India	[72]
*C. aquatica*	Solitary	Obclavate to cylindrical	Pale brown	34–46 × 3–4.5	(1–)2(–3)	On decaying leaves submerged in stream, Mexico	[24]
*C. arctespora*	Solitary or catenate	Cylindrical to obclavate	Brown to pale brown	13–63 × 4–7	2–20	On twigs of *Vaccinium*, USA	[73]
*C. asclepiadacearum*	Mostly solitary	Obclavato-cylindric to cylindrical	Pale olivaceous brown	44–192 × 10–25	Up to 26	On leaves of *Cryptolepis buchananii*, India	[74]
*C. azadirachtiana*	Solitary or catenate	Obclavate	Pale yellow	32–303.5 × 7–21.5	1–20	On leaves of *Azadirachta indica*, India	[75]
*C. barleriicola*	Solitary	Obclavate to cylindrical	Olivaceous yellow	41–246 × 10–18.5	3–14	On leaves of *Barleria cristata*, India	[75]
*C. bdellomorpha*	Solitary	Obclavate	Mid to dark-reddish brown	90–138 × 12–17	12–19	On dead stems of *Chusquea valdiviensis*, Chile	[26]
*C. beilschmiediae*	Solitary	Obclavate	Pale brown to brown	52–144.5 × 8.5–11	7–19	On dead branches of *Beilschmiedia intermedia*, China	[76]
*C. bombacearum*	Solitary or catenate	Obclavato-cylindrical to cylindrical	Pale to mid-olivaceous	26–206 × 8.5–17	Up to 15	On leaves of *Bombax malabaricum*, India	[77]
*C. bombacina*	Solitary or catenate	Obclavate to cylindrical	Light olivaceous	45–180 × 10–16	5–15	On living leaves of *Bombax ceiba*, India	[78]
*C. calicioidea*	Solitary	Obclavate	Subhyaline to pale golden brown	50–170 × 10–15	6–21	On wood, Sri Lanka	[79]
*C. carrisae*	Solitary	Obclavate to cylindrical	Olivaceous to very light brown	75–242 × 6–14	4–17	On leaves of *Carissa spinarum*, India	[80]
*C. caryotae*	Solitary	Obclavate to elongate	Pinkish brown	45–120 × 6–10	Up to 18	On dead rachis of *Caryota mitis*, Singapore	[81]
*C. cassiae*	Solitary	Obclavate	Pale brown to olivaceous brown	107.5–214 × 11–14	10–21	On dead branches of *Cassia surattensis*, China	[76]
*C. cassiicola*	Solitary or catenate	Obclavate to cylindrical	Subhyaline to pale olivaceous brown	40–220 × 9–22	4–20	On leaves of *Cassia*, Cuba	[5]
*C. catenulata*	Solitary or catenate	Obclavate to obclavato-cylindrical	Dark olivaceous yellow to pale olivaceous brown	27.5–225.5 × 11–19	1–24	On leaves of *Clerodendrum indicum*, India	[75]
*C. catharanthicola*	Solitary or catenate	Cylindrical	Brown	140–310 × 5.5–11	4–25	On leaves of *Catharanthus roseus*, China	[82]
*C. celastri*	Solitary	Obclavate to obclavato-cylindrical	Olivaceous to very light brown	55–120 × 8–15	7–17	On living leaves of *Celastrus paniculatus*, India	[83]
*C. chinensis*	Catenate	Obclavate	Pale brown	31–61 × 5–7.5	1–5	On dead branches of *Angiospermae*, China	[14]
*C. citricola*	Solitary or catenate	Cylindrical to obclavate	Subhyaline	48–150 × 4.5–8	4–18	On leaves of *Citrus aurantiifolia*, Australia	[79]
*C. clerodendrigena*	Solitary or catenate	Obclavate to cylindrical	Light olivaceous	60–220 × 16–22	3–13	On leaves of *Clerodendrum viscosum*, India	[84]
*C. colebrookiana*	Solitary or catenate	Obclavate, rarely cylindrical	Pale yellow	45–330 × 6–22	4–16	On leaves of *Colebrookea oppositifolia*, India	[75]
*C. combreli*	Solitary	Obclavate, rostrate	Pale olivaceous brown to olivaceous brown	40–122 × 8–11	4–10	On dead branches of *Combretum zeyheri*, Zambia	[85]
*C. cubensis*	Solitary or catenate	Cylindrical to obclavate	Pale brown to dark rusty brown	40–80 × 8–12	6–15	On dead petiole of *Coccothrinax*, Cuba	[86]
*C. cucurbiticola*	Solitary or catenate	Obclavato-cylindrical	Subhyaline to pale olivaceous	38.5–230 × 6.5–20	6–23	On leaves of *Coccinia grandis*, Nepal	[87]
*C. curvispora*	Solitary or catenate	Narrow obclavate	Straw-colored to mid-brown	40–250 × 10–12	5–10	On fallen herbaceous stems, USA	[88]
*C. doipuiensis*	Solitary	Obclavate to cylindrical	Subhyaline to moderately brown	136–165 × 5–25.5	–	On dead herbaceous branches, Thailand	[12]
*C. donacis*	Solitary	Obclavate	Olivaceous brown	45–70 × 8–12	10–14	On dead branches of *Donax*, China	[89]
*C. elaeidicola*	Solitary or catenate	Cylindrical to obclavate	Subhyaline or pale olivaceous brown	43–65 × 4–7	3–7	On dead leaves of *Elaeis guineensis*, Malaysia	[27]
*C. encephalarti*	Solitary	Obclavate	Medium olivaceous brown	100–150 × 11–15	1–12	On leaves of *Encephalartos*, South Africa	[90]
*C. eranthemi*	Solitary	Obclavate	Brown to pale olivaceous brown	65–176 × 11–14	5–25	On leaves of *Eranthemum wattii*, Singapore	[32]
*C. erythropsidis*	Solitary	Ellipsoid, doliiform to broad clavate	Pale brown to olivaceous brown	25–31 × 9–12	4	On dead branches of *Erythropsis colorata*, China	[91]
*C. euphorbiacearum*	Solitary or catenate	Obclavate	Subhyaline to light olivaceous brown	59–235 × 11–22.5	5–18	On leaves of *Manihot esculenta*, India	[71]
*C. euryae*	Solitary	Obclavate	Pale brown to brown	36–67 × 6–9	5–9	On dead branches of *Eurya inaequalis*, China	[92]
*C. fici-altissimae*	Solitary	Obclavate, rostrate	Dark brown	55–85 × 9–12	11–18	On dead branches of *Ficus altissima*, China	[89]
*C. fici-benjaminae*	Solitary	Obclavate	Pale olivaceous brown	51.5–71 × 8–11	5–10	On dead branches of *Ficus benjamina*, China	[76]
*C. ficigena*	Solitary	Obclavate to cylindrical	Light olivaceous brown	90–165 × 9–20	7–13	On leaves of *Ficus religiosa*, India	[93]
*C. flagellata*	Solitary	Obclavate, rostrate, smooth or verrucose	Dark brown	50–100 × 9–11	5–10	On wood of *Citrus*, Ghana	[94]
*C. fujianensis*	Solitary	Obclavate	Brown	31–90 × 6.5–10	4–10	On dead branches of *Myrioneuron faberi*, China	[95]
*C. gigaspora*	Solitary	Obclavate, rostrate	Pale to dark golden brown	100–270 × 19–28	9–52	On dead wood, Sri Lanka	[79]
*C. gorakhpurensis*	Solitary	Obclavate to ellipsoid	Pale olivaceous yellow	21–157 × 13–20	3–13	On leaves of *Erythrina indica*, India	[70]
*C. gracilis*	Solitary	Cylindric to obclavate	Olivaceous	92–138 × 5–7	10–22	On dead branches of *Piper betle*, Indonesia	[68]
*C. gymnocladi*	Solitary	Obclavate	Brown to dark brown	15–40 × 7–10.5	2–6	On dead branches of *Gymnocladus chinensis*, China	[92]
*C. hamata*	Solitary	Obclavate, hamatate at apex	Pale olivaceous brown	158–198 × 9–11	14–19	On dead wood, Indonesia	[68]
*C. hansfordii*	Solitary	Obclavate, rostrate	Straw-colored to brown	70–100 × 9–13	7–10	On dead wood, Uganda	[27]
*C. hemigraphidis*	Solitary	Obclavate	Pale olivaceous brown	72–218 × 12–15	5–25	On leaves of *Hemigraphis alternat*, Singapore	[32]
*C. heterospora*	Solitary	Cylindrical to obclavate	Pale olivaceous brown to olivaceous brown	75–170 × 6–20	6–12	On leaves of *Manihot utilissima*, Malaysia	[96]
*C. holopoteleae*	Solitary or catenate	Obclavato-cylindrical to cylindrical	Mid olivaceous	23–234 × 3.6–19.5	0–17	On leaves of *Holoptelea integrifolia*, India	[77]
*C. holopteleicola*	Solitary	Obclavate to obclavato-cylindrical	Olivaceous brown	33–148 × 5–20	0–11	On living leaves of *Holoptelea integrifolia*, India	[72]
*C. homaliicola*	Solitary	Obclavate, cylindrical	Subhyaline to straw-colored	110–220 × 11–22	13–28	On dead branches of *Homalium aylmeri*, Sierra Leone	[79]
“*C. ipomoeae*”	Solitary or catenate	Obclavate to cylindrical	Subhyaline to pale olivaceous	40–380 × 5–15	2–35	On leaves of *Ipomoea obscura*, India	[30]
*C. jasminicola*	Solitary	Obclavate	Pale olivaceous	39.5–176 × 10–21	2–18	On leaves of *Jasminum arborescens*, Nepal	[87]
*C. kamatii*	Solitary	Obclavate	Straw-colored	60–70 × 10–13	7–12	On dead twigs of *Vitis*, India	[69]
*C. kenyensis*	Solitary	Obclavate to obpyriform, rostrate	Subhyaline to pale brown	60–125 × 16–25	8–15	On dead stems of *Sericostachys scandens*, Kenya	[8]
*C. keskaliicola*	Solitary or catenate	Obclavato-cylindrical to cylindrical	Mid olivaceous	64–164 × 16–28	Up to 17	On leaves of *Hemidesmus indicus*, India	[74]
*C. laevistipitata*	Solitary	Broadly ellipsoid	Red–brown	17.5–24 × 7–8	(0–)1–2 (–3)	On *Pertusaria ophthalmiza* (lichen), USA	[97]
*C. lanneicola*	Solitary	Obclavate	Straw-colored to brown	40–58 × 10–15	4–5	On dead branches of *Lannea afzelii*, Sierra Leone	[79]
*C. lasianthi*	Solitary	Obclavate, sometimes rostrate	Pale brown to dark brown	50–103.5 × 8.5–10	4–8	On dead branches of *Lasianthus chinensis*, China	[76]
*C. leptoderridicola*	Solitary	Obclavate, rostrate	Subhyaline to straw-colored	70–120 × 14–17	6–16	On dead branches of *Leptoderris fasciculata*, Sierra Leone	[79]
*C. leucaenae*	Solitary	Obclavate, obovoid or ellipsoid	Pale yellow	16–298 × 10–19	1–28	On leaves of *Leucaena leucocephala*, India	[70]
*C. lignicola*	Solitary or catenate	Cylindrical	Subhyaline to pale brown	110–156 × 7–9	–	On submerged decaying wood, China	[12]
*C. ligustri*	Solitary or catenate	Obclavate to cylindrical	Straw-colored to brown	25–225 × 7.5–30	4–20	On leaves of *Ligustrum lucidum*, China	[98]
*C. litseae*	Solitary	Obclavate	Pale brown to olivaceous brown	105–235 × 10–12	14–34	On dead branches of *Litsea elongata*, China	[99]
*C. longispora*	Solitary	Cylindrical	Subhyaline to pale brown	120–330 × 5.5–8	11–24	On dead herbaceous stems, India	[100]
*C. maculiformis*	Solitary or catenate	Cylindrical to obclavate	Subhyaline to pale olivaceous brown	20–86 × 5–10	2–8	On rotten wood, Czech Republic	[101]
“*C. masseeanum*”	Solitary	Elongate to obclavate	Pale olivaceous	80–120 × 18–20	7–11	On branches of *Helicteres Isora*, India	[31]
*C. matuszakii*	Solitary or catenate	Cylindrical to obclavate	Pale brown to straw-colored or mid brown	56–260 × 10–12.5	2–10	On herbaceous stems of *Compositae*, USA	[102]
*C. merremiae*	Solitary or catenate	Cylindrical to obclavate	Pale olivaceous brown to pale brown	37–150 × 6–12.5	4–22	On leaves of *Merremia hirta*, China	[98]
*C. merrilliopanacis*	Solitary	Obclavate, rostrate	Straw-colored to brown	130–260 × 17–21	12–25	On dead branches of *Merrilliopanax listeri*, China	[61]
*C. micheliae*	Solitary	Obclavate, rostrate	Subyhaline to brown	333–360 × 15–19	12–28	On dead branches of *Michelia champaca*, China	[61]
*C. millettiae*	Solitary or catenate	Obclavate, smooth	Olivaceous brown to mid brown	30–182 × 7.5–14	2–15	On leaves of *Millettia*, China	[98]
*C. moracearum*	Solitary	Obclavate to cylindrical	Light olivaceous brown	27–163 × 12–20	5–16	On living leaves of *Ficus hispida*, India	[103]
*C. morindae-tinctoriae*	Solitary	Obclavate	Pale olivaceous	44–127 × 15–26.5	6–15	On leaves of *Morinda tinctoria*, India	[104]
*C. myrioneuronis*	Solitary	Obclavate	Pale brown to brown	30–46 × 6.5–8	3–4	On dead branch of *Myrioneuron faberi*, China	[92]
*C. mengsongensis*	Solitary	Obclavate to cylindrical, rostrate	Brown to golden brown	96–146 × 16.5–20.5	13–18	On dead branches, China	This study
*C. nana*	Solitary	Obclavate	Subhyaline to pale olivaceous brown	49.5–110 × 9–18.5	4–14	On leaves of *Lantana indica*, India	[104]
*C. nabanheensis*	Solitary	Obclavate to cylindrical, expanded to a rounded shape at the apex	Pale brown to brown	56–84 × 12–14	9–13	On dead branches, China	This study
*C. occidentalis*	Solitary	Ovoid to ellipsoidal	Subyhaline to pale brown	30–54 × 15–19	3–6	On leaves of *Cordia collococca*, Cuba	[105]
*C. palmicola*	Solitary	Obclavate to subcylindrical	Pale brown	40–70 × 6–9	5–7	On leaves of *Cocos australis*, Paraguay	[106]
*C. parapyrenariae*	Solitary	Obclavate	Pale brown to brown	70–100 × 11–14	5–9	On dead branches of *Parapyrenaria multisepala*, China	[99]
*C. parvispora*	Solitary	Ovoid	Brown	13–15 × 4.5–7.5	1–2	On dead twigs of *Gynotroches axillaris*, Singapore	[81]
*C. pedaliacearum*	Solitary or catenate	Obclavato-cylindrical to slightly acicular	Pale olivaceous	16–163 × 3.2–6	3–28	On leaves of *Sesamum indicum*, India	[107]
*C. peristrophicola*	Solitary	Obclavate to obclavato-cylindrical	Olivaceous to very light brown	60–135 × 5–16	5–12	On leaves of *Peristrophe bicalyculata*, India	[80]
*C. phylloshureae*	Solitary	Obclavate	Brown	30–50 × 8–10	6–10	On dead branches of *Phyllostachys sulphurea*, China	[89]
*C. pogostemonicola*	Solitary	Obclavate to obclavato-cylindrical	Olivaceous to olivaceous brown	77–288 × 8–14	5–24	On leaves of *Pogostemon plectrantoides*, India	[108]
*C. polyphragmia*	Solitary or catenate	Obclavate	Pale to mid golden brown	110–280 × 14–17	10–25	On decorticated branches of *Camellia japonica*, Japan	[109]
*C. pongamiicola*	Solitary	Obclavate, ellipsoidal, clavate or club-shaped	Light olivaceous yellow	18–65.2 × 8–16.5	1–6	On living leaves of *Pongamia pinnata*, India	[110]
*C. premnigena*	Solitary or catenate	Obclavate to obclavato-cylindrical	Subhyaline to pale yellow	52–265 × 10–15	1–19	On leaves of *Premna mucronata*, India	[75]
*C. proliferata*	Solitary or catenate	Obclavate, rostrate	Pale brown to brown	30–300 × 9–12	3–17	On wood of *Fagus sylvatica*, the Netherlands	[111]
*C. pruni*	Solitary or catenate	Obclavate	Olivaceous brown or brown	50–130 × 10–16	4–9	On bark of *Prunus serotina*, USA	[27]
*C. pseudocassiicola*	Solitary	Subcylindrical to obclavate	Medium brown	95–160 × 9–10	(4–)8–12(–17)	On leaves of *Byrsonima*, Colombia	[112]
*C. queenslandica*	Solitary	Obclavate	Pale brown	72–114 × 8–10	6–9	On phyllodes of *Acacia leiocalyx*, Australia	[15]
*C. rhapidis-humilis*	Solitary	Obclavate, rostrate	Pale brown to olivaceous brown	90–130 × 6–8	12–16	On dead branches of *Rhapis humilis*, China	[94]
*C. rhododendri*	Solitary	Obclavate to long rostrate	Pale brown to olivaceous brown	180–400 × 7.5–11	19–36	On dead branches of *Rhododendron hainanense*, China	[113]
*C. ripogoni*	Solitary	Obclavate	Brown	60–160 × 10–13.5	7–15	On dead stems of *Ripogonum scandens*, New Zealand	[9]
*C. rosacearum*	Solitary or catenate	Obclavate to obclavato-cylindrical	Subhyaline to pale olivaceous brown	26.5–269 × 9–18.5	1–18	On leaves of *Eriobotrya japonica*, India	[104]
*“C. ruelliae”*	Solitary	Obclavate	Brown to pale olivaceous brown	60–150 × 12–15	5–16	On leaves of *Ruellia macrophylla* and *Ruellia dipteracanthus*, Singapore	[32]
*C. sacchari*	Solitary	Obclavate, rostrate, verrucose or smooth	Pale brown to olivaceous brown	80–120 × 8–9	10–14	On dead branches of *Saccharum sinense*, China	[114]
*C. salasiae*	Solitary	Ellipsoidal, doliiform	Brown	17–20 × 8–12	0–2	On dead stems of grass, Cuba	[115]
*C. schleichericola*	Solitary or catenate	Obclavate	Pale olivaceous brown	22.5–66 × 3.8–8.5	1–12	On leaves of *Schleichera trijuga*, India	[107]
*C. scolopiae*	Solitary	Obclavate	Pale brown to brown	90–150 × 10–13	8–11	On dead branches of *Scolopia chinensis*, China	[116]
*C. sed-acaciae*	Solitary	Obclavate	Pale brown to olivaceous brown	40–70 × 11–13.5	8–12	On dead branches of *Acacia confusa*, China	[113]
*C. sidae*	Solitary	Obclavate to obclavato-cylindrical	Olivaceous brown to very light brown	25–220 × 7–17	2–24	On leaves of *Sida acuta*, India	[117]
*C. sinensis*	Catenate	Obclavate or fusiform, ellipsoid	Brown	21–42 × 8–9.5	3(–4)	On dead branches, China	[13]
*C. siwalika*	Solitary	Obclavate, rostrate	Pale straw-colored to golden brown	88–140 × 15–20	9–19	On branches of *Helicteres isora*, India	[109]
*C. smithii*	Solitary or catenate	Cylindrical	Subhyaline to golden brown	70–410 × 12–19	7–45	On bark of Ilex, UK	[79]
*C. solani*	Solitary or catenate	Obclavate to cylindrical	Olivaceous yellow	80.6–276 × 8–10	1–17	On leaves of *Solanum indicum*, India	[118]
*C. subcylindrica*	Catenate	Broadly ellipsoid, subcylindrical	Pale brown	18–60(–90) × 5–13	0–3(–6)	On leaves of *Lippia sidoides*, Brazil	[63]
*C. submersa*	Solitary or catenate	Obclavate, rostrate	Subhyaline to golden brown	100–150 × 16–24	9–13	On submerged decaying wood, China	[12]
*C. supkharii*	Solitary	Obclavate	Pale olivaceous brown	22.5–142.5 × 10–17.5	2–11	On leaves of *Phyllanthus parvifolius*, India	[119]
*C. tanaceti*	Solitary	Obclavate, smooth or verruculose	Pale brown to olivaceous brown	60–104 × 12–16	7–12	On dead branches of *Tanacetum vulgare*, China	[116]
*C. tectonae*	Solitary	Obclavate, rostrate, verrucose or smooth	Pale brown to olivaceous brown	110–160 × 10–12	12–18	On dead branches of *Tectona grandis*, China	[114]
*C. thailandica*	Mostly solitary	Obclavate	Brown	80–110 × 10–12	4–8	On wood, Thailand	[120]
*C. thorii*	Catenate	Subcylindrical, broadly ellipsoid to almost obovoid	Pale brown to medium olivaceous brown	20–30 × 5–7	(0–)1(–3)	On thallus, apothecia of *Lecanora*, Japan	[121]
*C. titarpaniensis*	Solitary	Obclavate to cylindrical	Olivaceous brown to light brown	50–340 × 5–20	5–35	On living leaves of *Lepidagathis*, India	[122]
*C. tomenticola*	Solitary	Cylindrical	Olivaceous brown to brown	50–230 × 10.5–20.5	3–6	On living leaves of *Terminalia tomentosa*, India	[110]
*C. toonae*	Solitary	Obclavate, rostrate	Pale brown to dark brown	65–144 × 7–9	4–14	On dead branches of *Toona sinensis*, China	[114]
*C. torulosa*	Solitary	Clavate	Dark olivaceous brown	35–60 × 13–20	3–5	On dead leaves of *Musa sapientum* Brazil	[123]
*C. tremae*	Solitary	Obclavate to obclavato-cylindrical	Light brown to brown	50–160 × 4–12	5–20	On dead petiole of *Trema orientalis*, India	[124]
*C. trematicola*	Solitary	Obclavate to ellipsoid	Pale olivaceous brown	104–296 × 11–16	1–12	On leaves of *Trema orientalis*, India	[118]
*C. trichiliae*	Solitary	Obclavate, rostrate	Subhyaline to straw-colored	53–74 × 9–11	4–6	On branches of *Trichilia heudelotii*, Sierra Leone	[27]
*C. trichoides*	Solitary	Obclavato-cylindrical or obclavate	Pale olivaceous brown	29–170 × 10–15	3–14	On leaves of *Triumfetta rhomboidea*, Nepal	[87]
*C. ulmacearum*	Solitary	Obclavate	Subhyaline to pale olivaceous brown	15–106 × 3.5–10	2–16	On leaves of *Trema orientalis*, India	[107]
*C. vismiae*	Solitary or catenate	Obclavate, rostrate	Pale olivaceous brown or straw-colored	55–107 × 6–9	3–5	On leaves of *Vismia guineensis*, Sierra Leone	[85]
*C. viticis*	Solitary or catenate	Cylindrical	Pale brown	80–383 × 6–9	–	On leaves of *Vitex rotundifolia*, China	[98]
*C. viticola*	Solitary or catenate	Obclavate, cylindrical to obovoid	Pale olivaceous brown	34–170 × 7–17.5	1–14	On leaves of *Cayratia carnosa*, India	[118]
*C. woodfordiana*	Solitary or catenate	Obclavate, rostrate	Light olivaceous brown	40–170 × 9.5–17	4–14	On leaves of *Woodfordia fruticosa*, India	[71]
*C. yerbae*	Solitary or catenate	Obclavate	Subhyaline to pale golden brown	72–170 × 16–18	8–19	On dead branches of *Ilex paraguayensis*, Argentina	[26]
*C. yunnanensis*	Solitary	Obclavato-cylindrical, rostrate	Brown to golden brown	80–128 × 16–19	3–16	On dead branches, China	This study
*C. ziziphae*	Solitary	Obclavato-cylindrical, cylindrical or clavate	Mid olivaceous brown to straw-colored	33–215 × 10–27	Up to 15	On leaves of *Ziziphus giraldii*, India	[77]

All conidia are smooth, except where indicated; 2“–”, the number of septation is not given.

*Kirschsteiniothelia* is one genus of many lignicolous fungi encountered in aquatic and terrestrial habitats. Following the treatment of Sun et al. [38], the genus currently consists of 39 species including *K. nabanheensis* [38,62,66], but most species have been identified based on morphological studies, and to date, only 17 species are represented by DNA sequences in GenBank. *Kirschsteiniothelia* has mainly been reported in the USA (nine species), China (eight species) and Thailand (six species), and little published information is has been recorded in other regions [38,62,66]. Thus, it is unclear whether is closely related with geographic regions.

Studies conducted to date on *Corynespora* and *Kirschsteiniothelia* have mainly focused on their alpha taxonomy, and most knowledge of both genera is related to woody and herbaceous hosts, whereas we have a less developed understanding of many natural substrates, such as dung, insects and other fungi, including lichens. Because most species of both genera lack cultures, some of them may have received scant consideration in single-spore isolation before the advent of Sanger sequencing and even have particular substrate requirements. Similarly, little attention has been accorded to the roles of these genera in decomposition and nutrient recycling, their geographical distribution, substrate specificities and teleomorph relationships. Therefore, it is not yet possible to quantify their roles in ecosystem function. Although this study broadens our understanding of the diversity of *Corynespora* and *Kirschsteiniothelia* taxa, additional large-scale surveys of fungal resources in aquatic and terrestrial habitats within different geographic regions and with different ecological environments, host information and climatic conditions are needed, which will contribute to a comprehensive knowledge of the fungal diversity of these genera. Further collaboration will also be necessary to quantify their functional roles and strengthen our ability to conserve fungal resources.

## Figures and Tables

**Table 1:** List of *Corynespora* species and GenBank accessions used in the phylogenetic analyses. New sequences are indicated in bold.

Taxon	Strain Number	GenBank Accession Numbers			
		SSU	LSU	ITS	*tef1-* *α*
*Corynespora cassiicola*	CBS 100822	GU296144	GU301808	–	GU349052
*C. citricola*	CBS 169.77	–	–	FJ852594	–
*C. doipuiensis*	MFLUCC 14–0022	MN648318	MN648326	MN648322	–
*C. encephalarti*	CBS 145555	–	MK876424	MK876383	–
*C. lignicola*	MFLUCC 16–1301	–	MN860554	MN860549	–
***C. mengsongensis***	**HJAUP C2000^T^**	**OQ060575**	**OQ060578**	**OQ060574**	–
***C. nabanheensis***	**HJAUP C2048^T^**	**OQ060576**	**OQ060580**	**OQ060577**	**OQ067526**
*C. pseudocassiicola*	CPC 31708	–	MH327830	MH327794	MH327877
*C. smithii*	L120	–	KY984297	KY984297	KY984435
*C. smithii*	L130	KY984419	KY984298	KY984298	KY984436
*C. smithii*	CABI 5649b	–	GU323201	FJ852597	GU349018
*C. smithii*	CBS 139925	–	KY984299	KY984299	–
*C. submersa*	MFLUCC 16–1101	–	MN860553	MN860548	–
*C. torulosa*	CBS 136419	–	MH877634	MH866095	–
*C. thailandica*	CBS 145089	–	MK047505	MK047455	MK047567
***C. yunnanensis***	**HJAUP C2132^T^**	**OQ060584**	**OQ060583**	**OQ060579**	–
*Periconia byssoides*	H 4600	AB797280	AB807570	LC014581	AB808546
*P. digitata*	CBS 510.77	AB797271	AB807561	LC014584	AB808537
*P. igniaria*	CBS 845.96	AB797277	AB807567	LC014586	AB808543
*P. macrospinosa*	CBS 135663	KP184080	KP184038	KP183999	–
*P. pseudodigitata*	KT 1395	NG_064850	NG_059396	NR_153490	AB808540
*Cyclothyriella rubronotata*	TR, CBS 121892	–	KX650541	KX650541	KX650516
*C. rubronotata*	TR9, CBS 141486	KX650507	KX650544	KX650544	KX650519

“–”, sequence is unavailable. Strain with T (ex-type). Abbreviations: **CABI:** International Mycological Institute, CABI-Bioscience, Egham, Bakeham Lane, U.K.; **CBS:** Central Bureau voor Schimmel cultures, Utrecht, The Netherlands; **CPC:** Collection of Pedro Crous housed at CBS; **HJAUP:** Herbarium of Jiangxi Agricultural University, Plant Pathology; **MFLUCC:** Mae Fah Luang University Culture Collection, Chiang Rai, Thailand; **SSU:** Small Subunit Ribosomal; **LSU:** Large Subunit Ribosomal; **ITS:** Internal Transcribed Spacer; ***tef1-α*:** Transcriptional Enhancer Factor 1-alpha; others are not registered abbreviations.

**Table 2:** List of *Kirschsteiniothelia* species and GenBank accessions used in the phylogenetic analyses. New sequences are in bold.

Taxon	Strain	Genbank Accession Numbers		
		ITS	LSU	SSU
*Acrospermum adeanum*	M133	EU940180	EU940104	EU940031
*A. compressum*	M151	EU940161	EU940084	EU940012
*A. gramineum*	M152	EU940162	EU940085	EU940013
*Anisomeridium ubianum*	MPN94	–	GU327709	JN887379
*Flavobathelium epiphyllum*	MPN67	–	GU327717	JN887382
*Kirschsteiniothelia aethiops*	CBS 109.53	–	AY016361	AY016344
*K. aethiops*	MFLUCC 16–1104	MH182583	MH182589	MH182615
*K. aethiops*	S–783	MH182586	MH182595	MH182617
*K. aethiops*	MFLUCC 15–0424	KU500571	KU500578	KU500585
*K. aquatica* ^T^	MFLUCC 17–1685	MH182587	MH182594	MH182618
*K. arasbaranica*	IRAN 2509C	KX621986	KX621987	KX621988
*K. arasbaranica* ^T^	IRAN 2508C	KX621983	KX621984	KX621985
*K. cangshanensis* ^T^	MFLUCC 16–1350	MH182584	MH182592	–
*K. fluminicola* ^T^	MFLUCC 16–1263	MH182582	MH182588	–
*K. lignicola* ^T^	MFLUCC 10–0036	HQ441567	HQ441568	HQ441569
***K. nabanheensis*** **^T^**	**HJAUP C2004**	**OQ023197**	**OQ023273**	**OQ023038**
***K. nabanheensis***	**HJAUP C2006**	**OQ023274**	**OQ023275**	**OQ023037**
*K. phoenicis* ^T^	MFLUCC 18–0216	MG859978	MG860484	MG859979
*K. rostrata* ^T^	MFLUCC 15–0619	KY697280	KY697276	KY697278
*K. rostrata*	MFLUCC 16–1124	–	MH182590	–
*K. submersa* ^T^	MFLUCC 15–0427	KU500570	KU500577	KU500584
*K. submersa*	S–481	–	MH182591	MH182616
*K. submersa*	S–601	MH182585	MH182593	–
*K. tectonae* ^T^	MFLUCC 12–0050	KU144916	KU764707	–
*K. thailandica* ^T^	MFLUCC 20–0116	MT985633	MT984443	MT984280
*K. thujina*	JF 13210	KM982716	KM982718	KM982717
*Megalotremis verrucosa*	MPN104	–	GU327718	JN887383
*Phyllobathelium anomalum*	MPN 242	–	GU327722	JN887386
*P. firmum*	ERP 3175	–	GU327723	–
*Pseudorobillarda eucalypti*	MFLUCC 12–0422	KF827451	KF827457	KF827463
*P. phragmitis*	CBS 398.61	MH858101	EU754203	EU754104
*Strigula guangxiensis* ^T^	HMAS-L0138040	NR_146255	MK206256	–
*S. nemathora*	MPN 72	–	JN887405	JN887389
*Tenuitholiascus porinoides* ^T^	HMAS-L0139638	–	MK206259	MK352441
*T. porinoides*	HMAS-L0139639	–	MK206258	MK352442
*T. porinoides*	HMAS-L0139640	–	MK206260	MK352443

“–”, sequence is unavailable; strain with T, ex type. Abbreviations: **CBS:** Central Bureau voor Schimmel cultures, Utrecht, the Netherlands; **HJAUP:** Herbarium of Jiangxi Agricultural University, Plant Pathology; **HMAS:** Fungarium-Lichenum of the Institute of Microbiology, Chinese Academy of Sciences; **IRAN:** Iranian Fungal Culture Collection, Iranian Research Institute of Plant Protection, Tehran, Iran; **MFLUCC:** Mae Fah Luang University Culture Collection, Chiang Rai, Thailand; **ITS:** internal transcribed spacer; **LSU:** large subunit ribosomal; **SSU:** small subunit ribosomal; others are not registered abbreviations.

## Data Availability

All sequences generated in this study were submitted to GenBank.
